# A feasibility study using quantitative and interpretable histological analyses of celiac disease for automated cell type and tissue area classification

**DOI:** 10.1038/s41598-024-79570-1

**Published:** 2024-12-02

**Authors:** Michael Griffin, Aaron M. Gruver, Chintan Shah, Qasim Wani, Darren Fahy, Archit Khosla, Christian Kirkup, Daniel Borders, Jacqueline A. Brosnan-Cashman, Angie D. Fulford, Kelly M. Credille, Christina Jayson, Fedaa Najdawi, Klaus Gottlieb

**Affiliations:** 1 PathAI, Inc., 1325 Boylston Street, Suite 10000, Boston, MA 02215 USA; 2 Eli Lilly and Company, Indianapolis, IN USA

**Keywords:** Artificial intelligence, Celiac disease, Histology, Machine learning, Coeliac disease, Machine learning

## Abstract

Histological assessment is essential for the diagnosis and management of celiac disease. Current scoring systems, including modified Marsh (Marsh–Oberhuber) score, lack inter-pathologist agreement. To address this unmet need, we aimed to develop a fully automated, quantitative approach for histology characterisation of celiac disease. Convolutional neural network models were trained using pathologist annotations of hematoxylin and eosin-stained biopsies of celiac disease mucosa and normal duodenum to identify cells, tissue and artifact regions. Biopsies of duodenal mucosa of varying celiac disease severity, and normal duodenum were collected from a large central laboratory. Celiac disease slides (*N* = 318) were split into training (*n* = 230; 72.3%), validation (*n* = 60; 18.9%) and test (*n* = 28; 8.8%) datasets. Normal duodenum slides (*N* = 58) were similarly divided into training (*n* = 40; 69.0%), validation (*n* = 12; 20.7%) and test (*n* = 6; 10.3%) datasets. Human interpretable features were extracted and the strength of their correlation with Marsh scores were calculated using Spearman rank correlations. Our model identified cells, tissue regions and artifacts, including distinguishing intraepithelial lymphocytes and differentiating villous epithelium from crypt epithelium. Proportional area measurements representing villous atrophy negatively correlated with Marsh scores (*r* =  − 0.79), while measurements indicative of crypt hyperplasia positively correlated (*r* = 0.71). Furthermore, features distinguishing celiac disease from normal duodenum were identified. Our novel model provides an explainable and fully automated approach for histology characterisation of celiac disease that correlates with modified Marsh scores, potentially facilitating diagnosis, prognosis, clinical trials and treatment response monitoring.

**Supplementary Information:**

The online version contains supplementary material available at 10.1038/s41598-024-79570-1.

## Introduction

Celiac disease, an autoimmune disease triggered by dietary gluten, affects around 1% of the population^1^. Its diagnosis can be challenging due to symptom diversity, spanning from no symptoms to severe malabsorption^1,2^. Patients with celiac disease face a slightly increased overall risk of developing bowel lymphoma in comparison with the general population^2^. Histological assessment is crucial for the diagnosis and management of celiac disease^3^, as well as for endpoint assessment in clinical trials^4^, with findings of intraepithelial lymphocytosis, crypt hyperplasia, and villous atrophy indicative of the presence of the disease^5^. Clinical evaluations often rely on assessments of disease activity, demonstrated by changes in histology and characterised according to disease severity by classification systems such as the modified Marsh (Marsh–Oberhuber) score and Corazza-Villanacci classification^3,6,7^, or morphometric approaches that measure villus height, crypt depth, and changes in the villus height to crypt depth ratio^8,9^. The US Food and Drug Administration recommends using a clinically accepted histological scale such as the Marsh score for screening samples in clinical studies of treatments for celiac disease, to ensure patient eligibility at enrolment. Furthermore, histology is also recommended as a co-primary endpoint in these studies^10^. However, because inter-observer agreement is low for metrics that rely on qualitative assessments^6^, the Tampere task force recommends using histological evaluations that employ quantitative measurements, e.g., villous height/crypt depth ratio and percent change in intraepithelial lymphocyte density^11^.

Celiac disease is often underdiagnosed due to variation between pathologists in their assessment of biopsy tissue samples^12^, even if multiple biopsies are obtained^3,5^. Poor quality of biopsy tissue and overlapping histopathology features between related conditions may contribute to this variability^5,12,13^. Recently, there has been increased interest in applying machine learning (ML) to pathology assessments^14,15^, including to improve the accuracy and efficiency of celiac disease diagnosis^16^.

Such automation is expected to significantly reduce variability^16,17^, enabling smaller clinical studies to attain sufficient statistical power and demonstrate treatment effects. Indeed, convolutional neural network (CNN) tissue and cell model predictions from gastrointestinal samples have been used to create human interpretable features (HIFs) that enable the quantitative assessment of inflammatory pathological changes in non-celiac gastrointestinal diseases^18^. The application of ML to diagnosis has also been examined for inflammatory bowel disease^19^.

Although previous research has successfully employed ML to detect celiac disease and assess disease severity based on Marsh scores^17^, this study aims to bridge critical gaps in the current research landscape. The work presented here represents the first report of an ML application for celiac disease that provides fully explainable tissue segmentation and cell classifications across whole slide images (WSIs) of duodenal mucosal biopsies. Through this approach, we have enabled the extraction of HIFs, such as cell densities, cell count proportions, and tissue area proportions, all of which exhibit correlations with Marsh scores. By utilising ML-based quantification, this study aims to objectively and comprehensively characterise celiac disease histology, address the limitations of manual histological assessments, and provide granular data for translational research and clinical trials. We believe such an approach has tremendous potential as a scalable tool for measuring disease severity and monitoring treatment response.

## Materials and methods

### Data set characteristics

WSIs of hematoxylin and eosin (H&E)-stained biopsies of duodenal mucosa of varying celiac disease severity (*N* = 318) and mucosa of normal duodenum (*N* = 58) were collected from PathAI Diagnostics (Memphis, TN, USA) (Supplemental Fig. 1). Tissues were originally embedded to optimise orientation, and orientation of villi was included as a quality control metric.

**Fig. 1:**
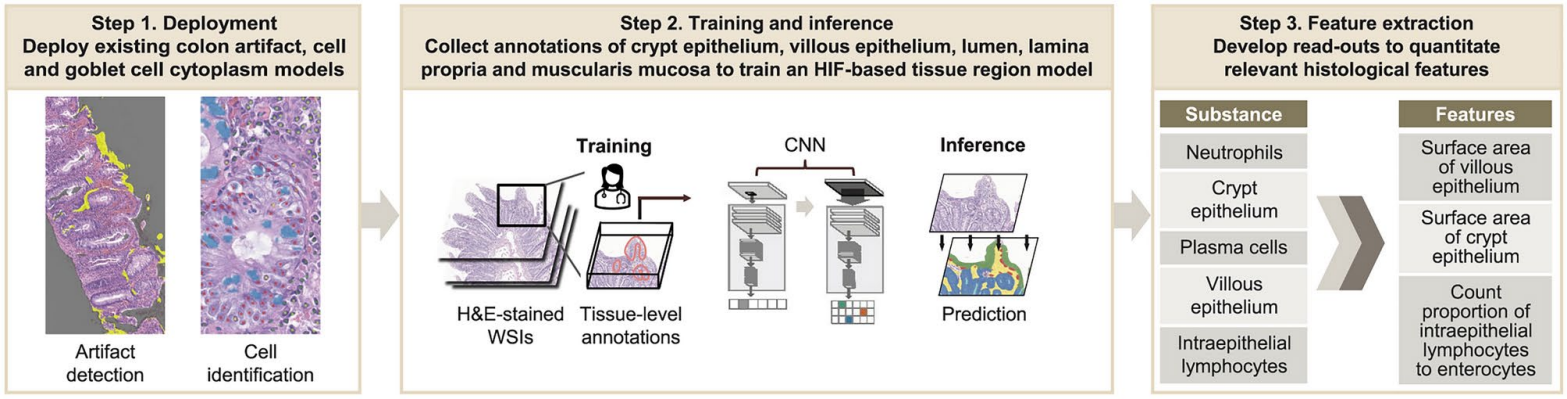
Proof-of-concept development of models based on HIFs on training data. CNN, convolutional neural network; H&E, hematoxylin and eosin; HIF, human interpretable feature; WSI, whole slide image.

The cohort size was determined based on the project’s scope and the availability of small intestine biopsies encompassing the full spectrum of celiac disease histology at the central laboratory (PathAI Diagnostics, Memphis, TN, USA). Slides were scanned at 40 × objective magnification using the Aperio GT450 slide scanner (Leica Biosystems, Wetzlar, Germany). Celiac disease slides were split into training (*n* = 230; 72.3%), validation (*n* = 60; 18.9%) and test (*n* = 28; 8.8%) datasets to ensure the even distribution of available patient metadata. For normal duodenum, slides were divided into a similar ratio of training (*n* = 40; 69.0%), validation (*n* = 12; 20.7%) and test (*n* = 6; 10.3%) datasets.

### Patient involvement

The scope of the study concentrated on the analysis of digital pathology images and, as such, direct patient involvement was not a component of the research design, implementation or dissemination plans.

### ML-based tissue model development

We developed a model to identify and quantify relevant tissue regions, and we also utilised a previously trained model^18^ to identify and quantify cell types and artifact regions on H&E-stained WSIs of celiac disease and normal duodenum (Fig. 1). Using these identified cells and tissue regions, histological features relevant to celiac disease and representing surrogate measures of modified Marsh score components were quantified, including the proportion of intraepithelial lymphocytes to enterocytes in villous epithelium and the surface areas of villous epithelium and crypt epithelium. The latter two features assess villous height and crypt hyperplasia, respectively.

WSIs were annotated by board-certified gastrointestinal pathologists. In total, 8356 tissue region annotations were collected. Annotations of crypt epithelium, villous epithelium, crypt lumen, lamina propria, blood vessels, muscularis mucosa and other tissue (including Brunner’s glands and submucosa) were used to train a HIF-based tissue segmentation model. From these annotations, a CNN model was trained to produce pixel-level predictions of small intestinal mucosa tissue regions. Previously developed models to detect and exclude tissue artifacts and identify and classify the cells in colon tissue were also deployed^18^. Tissue and cell model predictions were visualised as heatmaps on WSIs. Heatmap transformations were used to remove artifact regions (e.g., debris, tissue folds, out-of-focus regions), extracting features only from high-quality tissue.

### Validation and review of cell and tissue models

A PathAI pathologist (FN) performed quality control of the tissue labels used for model training and qualitatively reviewed the tissue and cell overlays representing model predictions on H&E-stained WSIs. This qualitative review helped guide the iterative model development (Supplemental Fig. 2a). Representative overlays for normal duodenum, mild villous blunting, and severe villous blunting are shown in Supplemental Fig. 2b.

**Fig. 2:**
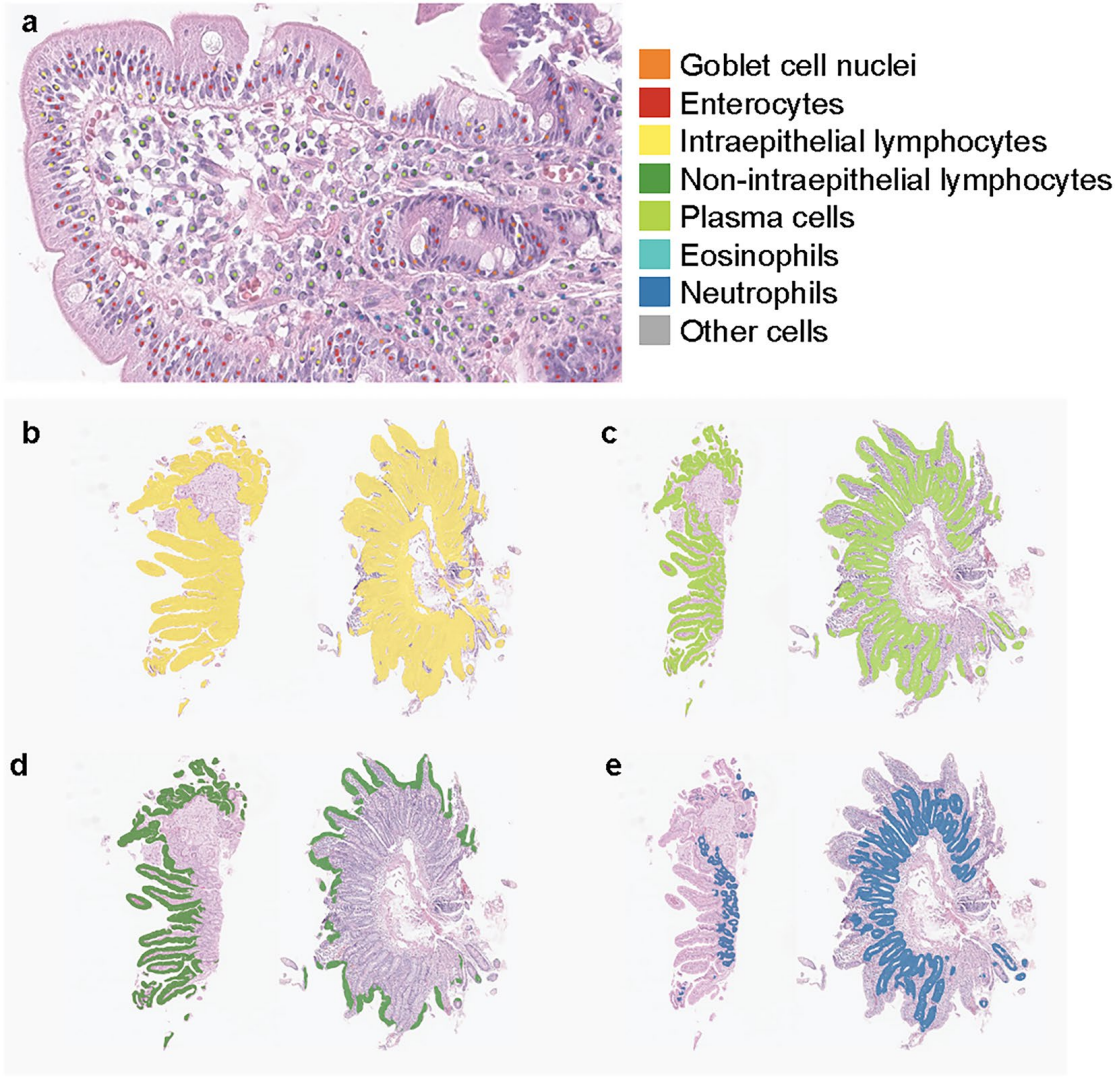
Cell and tissue segmentation model overlays showing distinct tissue regions. (**a**) Overlays generated by cell segmentation model for model deployment. (**b**) Overlay showing normal duodenum (left) vs. celiac disease (right) in mucosa. (**c**) Overlay showing normal duodenum (left) vs. celiac disease (right) in all epithelia. (**d**) Overlay showing normal duodenum (left) vs. celiac disease (right) in villous epithelium. (**e**) Overlay showing normal duodenum (left) vs. celiac disease (right) in crypt epithelium.

To establish ground truth for cell model prediction accuracy, representative image frames were sampled (75 μm × 75 μm; *N* = 160). Frames were exhaustively annotated for all model-predicted cell types by five gastrointestinal pathologists. The guidelines for the differentiation of plasma cells, lymphocytes, neutrophils, and eosinophils from H&E-stained images were as follows. Plasma cells were to be identified as cells with an eccentrically placed nucleus with a “clock-face” chromatin pattern, abundant basophilic cytoplasm, and a Golgi apparatus visible as a pale, perinuclear “halo.” Lymphocytes were to be identified as cells with a large, round nucleus with densely clumped chromatin taking up the majority of the cell, scant cytoplasm appearing as a blue rim around the nucleus, and small, uniform overall size. Neutrophils were to be identified as cells with a multi-lobed nucleus connected by thin chromatin strands, a light pink cytoplasm with fine, pale granules, a larger size than a lymphocyte. Eosinophils were to be identified as cells with a bi-lobed or segmented nucleus with consistent spectacled appearance, a cytoplasm filled with large, bright eosinophilic granules. Hierarchical clustering was performed on these annotations and model predictions as previously described to identify cell locations^18^. To account for potential pathologist bias and variability, Bayesian-estimated ground truths were used to quantify and compare the performance of the annotators and the model (Supplemental Fig. 3).

**Fig. 3:**
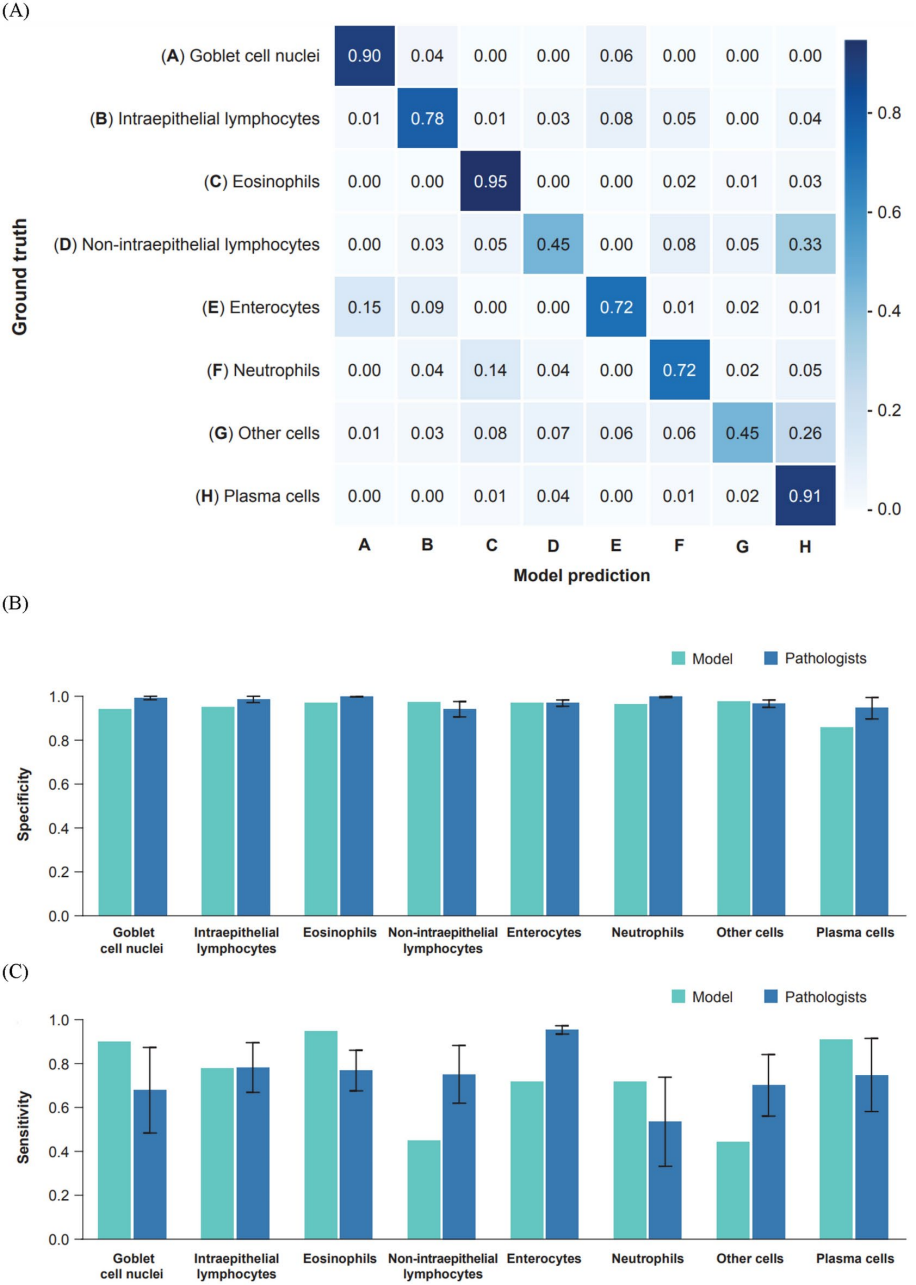
(**A**) Cell model confusion matrix showing sensitivity across different cell types. (**B**) Accuracy of cell model predictions compared with pathologists by specificity. (**C**) Accuracy of cell model predictions compared with pathologists by sensitivity.

### Evaluation of model-derived HIFs

HIFs (e.g., the proportional area of villous epithelium relative to lamina propria) were extracted from WSIs of normal duodenum (*N* = 52) and scored celiac disease (*N* = 118). HIFs were correlated with modified Marsh scores provided by a single gastrointestinal pathologist (type 0, normal lesions; type 1, infiltrative lesions; type 2, hyperplastic lesions; and types 3a, 3b and 3c, destructive lesions)^6^ using Spearman rank correlations. After establishing correlations between HIFs and modified Marsh scores, potential differences in the model-derived features between celiac disease and normal duodenum were evaluated.

### Statistical analysis

To assess cell model performance, sensitivity and specificity were calculated for both the cell model predictions and each pathologist annotation compared with the consensus on representative image frames. To evaluate the model-generated HIFs, each HIF was assessed for correlation with a single pathologist read of the modified Marsh scores using Spearman rank correlations. Data analyses in this study used the programming language Python (OpenEDG Python Institute, West Pomerania, Poland) for tissue and cell model development. Additionally, OpenSlide Python (Carnegie Mellon University, Pittsburgh, PA, USA) was used to load WSIs, Matplotlib (John D Hunter, Matplotlib Development Team and NumFOCUS, Austin, TX, USA) was used for plotting graphs, and PyTorch (PyTorch Foundation, the Linux Foundation, San Francisco, CA, USA) was used for tissue and cell model development. To associate model-derived features of celiac disease following correlations with modified Marsh scores, mean (standard deviation) feature levels were used to show differences between celiac disease and normal duodenum. *P* values were calculated by independent *t*-test.

## Results

### Model development for quantitation of celiac disease histological features

The tissue model developed herein, as well as previously trained cell and artifact models^18^, were deployed on H&E-stained WSIs of celiac disease and normal duodenum. Relevant cell types identified included neutrophils, plasma cells, enterocytes, intraepithelial lymphocytes, non-intraepithelial lymphocytes, eosinophils and goblet cells (Fig. 2); all other cell types are predicted as “other cells”. In addition, tissue regions identified included villous epithelium, crypt epithelium, lamina propria, muscularis mucosa, and blood vessels. Tissue regions such as total epithelium and mucosa could also be extracted from the tissue segmentation overlays. The tissue model distinguished villous epithelium from crypt epithelium.

The cell model’s performance was evaluated by comparing it with consensus pathologists’ annotations using Bayesian-estimated ground truths. Here, we sought to concentrate this validation on overlapping cells, focusing on cell confusion. The cell model demonstrated acceptable sensitivity for most cell types (Fig. 3a). To determine model accuracy, labels from five gastrointestinal pathologists (Supplemental Fig. 3a) were compared to cell model predictions on representative image frames, as described in Supplemental Fig. 3b. We reported elements of the F1 score for both cell model predictions and pathologists’ labels for each of the cell types (Fig. 3b,c). Overall, cell model specificity remained relatively consistent and was similar to that of the pathologists for most cell types; a slight difference was observed for plasma cells, whereas sensitivity was more variable outside the intraepithelial lymphocyte class.

### Correlation of surrogate features with modified Marsh score

**Fig. 4:**
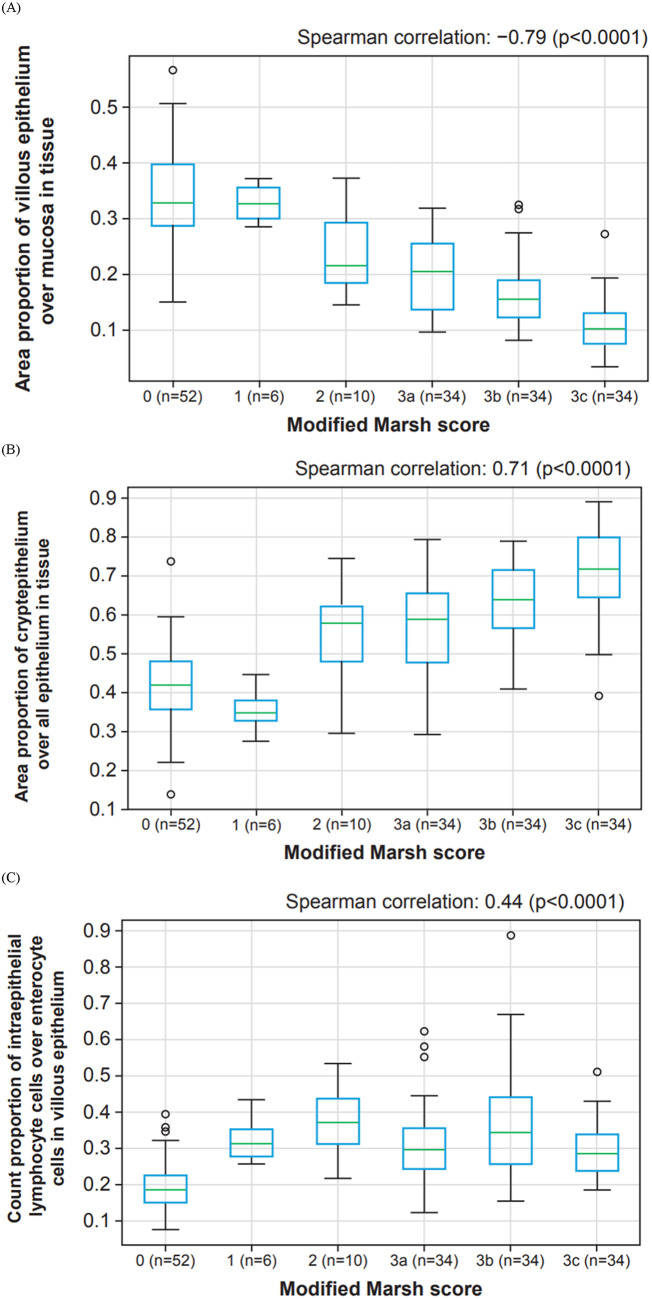
Example cell and tissue segmentation model correlation with modified Marsh score. (**A**) Surrogate features of villous blunting. (**B**) Surrogate features of crypt hyperplasia. (**C**) Surrogate features of intraepithelial lymphocyte infiltration.

HIFs from our models were analyzed to assess correlation with modified Marsh scores, which were provided by a single gastrointestinal pathologist. The area of villous epithelium relative to mucosa was negatively correlated with modified Marsh score (Spearman *r* =  − 0.79, *p* < 0.0001) (Fig. 4a). The area of crypt epithelium in tissue (Fig. 4b) positively correlated with modified Marsh score (Spearman *r* = 0.71, *p* < 0.0001), as did the number of intraepithelial lymphocyte cells relative to enterocyte cells in villous epithelium (Fig. 4c) (Spearman *r* = 0.44, *p* < 0.0001). These results are summarised in Supplemental Table 1.

**Table 1:** Association of model-derived features with celiac disease.

		Normal duodenum	Celiac disease	
	Feature	Mean (SD)	Mean (SD)	*P* value
Features quantifying villous atrophy	Area proportion of villous epithelium over mucosa in tissue	0.33 (0.08)	0.15 (0.07)	< 0.0001
	Area proportion of villous epithelium over all epithelium in tissue	0.58 (0.10)	0.36 (0.14)	< 0.0001
	Area proportion of villous epithelium over lamina propria in tissue	1.11 (0.35)	0.36 (0.23)	< 0.0001
Features quantifying crypt hyperplasia	Area proportion of crypt epithelium over usable tissue	0.21 (0.05)	0.23 (0.07)	0.12
	Area proportion of lamina propria over crypt epithelium in tissue	1.38 (0.49)	1.90 (0.82)	< 0.0001
	Area proportion of crypt epithelium over all epithelium in tissue	0.42 (0.10)	0.64 (0.14)	< 0.0001
	Area proportion of crypt epithelium over mucosa in tissue	0.24 (0.05)	0.27 (0.07)	< 0.01
Surrogate features for villous height/crypt depth ratio	Area proportion of villous epithelium over crypt epithelium in tissue	1.54 (0.87)	0.64 (0.47)	< 0.0001
Features quantifying intraepithelial lymphocyte infiltration	Count proportion of intraepithelial lymphocytes over enterocytes in villous epithelium	0.20 (0.07)	0.31 (0.11)	< 0.0001
	Density of intraepithelial lymphocytes in villous epithelium	910.27 (303.15)	1446.27 (463.91)	< 0.0001
Features quantifying expansion of inflammatory cells in lamina propria	Count proportion of plasma cells over all cells in lamina propria	0.23 (0.05)	0.29 (0.10)	< 0.001
	Density of plasma cells in lamina propria	2131.66 (593.54)	2725.74 (1171.97)	< 0.001
	Density of lymphocytes in lamina propria	2483.02 (793.40)	1808.05 (641.31)	< 0.0001
	Count proportion of lymphocytes over all cells in lamina propria	0.27 (0.06)	0.19 (0.06)	< 0.0001
	Area proportion of lamina propria over mucosa in tissue	0.31 (0.04)	0.47 (0.08)	< 0.0001
	Total number of cells in lamina propria	54013.02 (28142.69)	89,593.13 (50,629.86)	< 0.0001
Other features quantifying inflammatory cells	Count proportion of neutrophils over all cells in mucosa	0.03 (0.01)	0.05 (0.02)	< 0.0001
	Count proportion of eosinophils over all cells in mucosa	0.02 (0.01)	0.03 (0.01)	< 0.0001

SD, standard deviation.

In addition, the HIFs extracted from the cell and tissue models were also used to evaluate features that were distinguished between normal biopsies from those with celiac disease. For example, the proportional area of villous epithelium relative to mucosa and the proportional area of villous epithelium relative to crypt epithelium were both lower in celiac disease tissue compared with normal tissue, while the proportional area of crypt epithelium relative to total epithelium, the proportional area of lamina propria over mucosa, and the density of intraepithelial lymphocytes in villous epithelium were higher in celiac disease (*p* < 0.0001 for all comparisons) (Table 1).

## Discussion

Histological assessment of celiac disease plays a crucial role in diagnosing disease and evaluating the effectiveness of clinical interventions^3^. However, inter-observer variability can affect the consistency and accuracy of results^6^. To overcome this limitation and augment pathologists’ assessments of disease severity, we aimed to develop a fully automated and explainable approach to quantify the cellular and tissue-based features of celiac disease in H&E-stained clinical samples. The HIFs extracted from this model reflected histological changes that were measured by modified Marsh scores, potentially providing a quantitative and reproducible means to assess celiac disease severity.

Our model produced continuous feature measurements that can be interpreted as surrogate markers of celiac disease pathology (Supplemental Table 1). The relationship of these features with the ordinal Marsh score categories can be used as a benchmark to measure the model’s performance. For example, we examined the area of villous epithelium relative to the area of mucosa as an indicator of villous blunting, a hallmark of celiac disease, and found a negative correlation with higher modified Marsh scores. To gauge crypt hyperplasia, a more subtle feature, we examined the area of crypt epithelium relative to total epithelial area, revealing a positive correlation between this feature and Marsh scores of 2 and above. The trained cell model directly quantitated the proportion of intraepithelial lymphocytes relative to the number of enterocytes within the villous structures. As expected, these values increased with disease severity. The lower strength of this specific correlation could be attributed to how the modified Marsh score is calculated, in which pathologists assess the presence of > 30 intraepithelial lymphocytes per 100 enterocytes when differentiating scores 0 from 1 rather than quantifying any further increase in intraepithelial lymphocytes as the disease severity increases. In addition, the features extracted from our model align with other clinical trial endpoints such as measurement of villous height and crypt depth^4,8^. Existing celiac disease scoring systems, such as the modified Marsh score, primarily rely on qualitative and descriptive categorisations, leading to subjectivity and limited sensitivity to subtle changes^20^. In this study, we propose an alternative approach, utilising ML techniques to enable continuous, quantitative evaluation of the histological changes in celiac disease. By capturing histological alterations on a granular and objective scale, this novel approach offers enhanced sensitivity to changes in intraepithelial lymphocyte density, as well as villous and crypt epithelial surface area, overcoming limitations of the qualitative assessments of conventional manual scoring systems. It is worth noting that there are other scoring systems apart from the Marsh classification for celiac disease, including the Corazza-Villanacci classification^7^. Future studies to apply digital pathology algorithms to these additional scoring approaches in celiac disease are warranted.

Notably, some of our model-extracted HIFs directly quantified features of intraepithelial lymphocytes. This cell type is an essential consideration during disease assessment, as the presence of > 30 intraepithelial lymphocytes per 100 enterocytes in the duodenum is a defining feature of celiac disease^21^. The HIFs extracted from our model include count proportions and/or density of intraepithelial lymphocytes, specifically in the villous epithelium. This model also allowed for the extraction of features relating to intraepithelial lymphocytes in crypt epithelium and a comparison of their density in villous and crypt epithelium, providing a comprehensive overview of the spatial distribution of this cell type within distinct epithelial regions. Additional relevant features included the proportional area of villous epithelium (quantifying the change related to villous atrophy), the proportional area of crypt epithelium (quantifying crypt hyperplasia) and the ratio of villous epithelium area to crypt epithelium area (quantitatively capturing the relationship of villous height to crypt depth)^22^. A decreased epithelial surface area is a long-recognised feature of celiac disease^23^. Altered crypt surface area represents a histological change that results in symptoms of celiac disease, e.g., malabsorption. While this metric cannot be directly measured by manual pathology approaches, it has been proposed that alternative measurements, such as villous height and crypt length, have the potential to act as surrogate features allowing for villous surface area to be estimated^24^. Indeed, decreased villous area has previously been associated with increased Marsh score^25^. The villous and crypt features extracted by our model have the potential to further inform a clinical estimation of small intestine epithelial surface area in celiac disease. This promising methodology should be further investigated as a quantitative approach to measuring the histological features of celiac disease, augmenting traditional approaches to biopsy assessment in this setting.

The key strengths of this study become apparent when considering that these model-generated features not only bear relevance to the modified Marsh scoring system, but are also essential components of the histological hallmarks of celiac disease (Table 1)^22^. These HIFs encompass features not previously incorporated into any formalised scoring system, such as relative numbers and density of inflammatory cells (including lymphocytes, plasma cells, eosinophils and neutrophils) in lamina propria or in mucosa. These metrics characterise the immune microenvironment within celiac biopsies, as well as the total area and area proportion of lamina propria, capturing the expansion of lamina propria, a phenomenon known to be associated with disease activity^22^. Furthermore, one of the key strengths of this study lies in our model’s capacity to discern between normal duodenum and celiac disease through the quantification of features associated with the disease microenvironment in mucosal biopsies. As expected, quantifying features of villous atrophy, as evidenced by reduced area proportion of this feature, and the augmented area proportion of crypt epithelium signifying mucosa crypt hyperplasia, distinguished between the histology of unaffected biopsies and those indicative of celiac disease. Supplementary quantitative attributes of the inflammatory microenvironment known to be associated with celiac disease, encompassing the infiltration of chronic inflammatory cells like lymphocytes and plasma cells within the lamina propria, coupled with the associated expansion of this layer^22^, further distinguished normal biopsy samples from those with celiac disease. Discernible differences between the two groups were also observed in the quantitative evaluation of granulocytes, which has been previously described^26,27^. Finally, a key, novel aspect of this study is our model’s ability to predict cell types from an H&E-stained WSI. Prior studies have facilitated intraepithelial lymphocyte and lamina propria B cells detection and quantification in celiac disease tissue^28,29^. However, these studies relied on immunohistochemical staining to detect the cells of interest. The capability to quantify celiac disease features on H&E-stained images, which are routinely used for biopsy assessment, has the potential to streamline the evaluation of clinical celiac disease specimens. While our model was limited by the small sample size, additional assessment involving larger cohorts will allow future refinement of the model’s performance. The cell model can also be trained specifically on duodenum biopsies and expanded to predict features associated with additional cell types, e.g., Paneth cells. An additional limitation of the current approach is related to the extraction of HIFs across a specific tissue area in the entire slide, which overlooks the potential variation in histological disease changes between different tissue fragments, as well as orientation variability. In a manual assessment of celiac disease in biopsies, pathologists often determine disease severity based on the most severely affected tissue region. To address this limitation, future work will focus on reporting HIFs separately for specific regions of interest within the tissue sample. This strategy is expected to allow for a more comprehensive and accurate assessment of disease severity within distinct tissue regions. Finally, our study did not account for orientation variability. The effect of tissue orientation on model-generated HIFs in celiac disease WSIs necessitates further study.

In conclusion, we foresee that ML-supported histological analysis will play a pivotal role in the advancement of precision medicine for patients with celiac disease. To our knowledge, this is the first report of fully explainable ML-based tissue and cell classifications across the WSIs of mucosal biopsies in celiac disease, enabling the extraction and statistical analysis of HIFs to empower translational research and clinical trials. The resulting quantitative model-generated HIFs can be used to build predictive models of existing Marsh scores or function as a continuous measurement, tracking histological change in celiac biopsies. Expanding upon this foundation, as we proceed to develop classification models aimed at predicting clinical outcomes alongside slide-level scores, we anticipate that the interpretability enabled by the utilisation of HIFs is poised to serve a dual purpose: validating the integrity of these models and revealing novel insights into disease biology. We believe that this ML-based assessment has potential as a scalable tool for measuring disease severity, risk stratification, prognostic evaluation, evaluating endpoints in clinical trials, and monitoring of treatment responses—ultimately advancing the care of patients with celiac disease.

## Supplementary Information

Supplementary Information.

## Data Availability

Model parameters for cell and tissue models, and codes for model training, inference and feature extractions are not disclosed. Access requests for such code will not be considered to safeguard PathAI’s intellectual property. All feature tables, as well as source code, for reproducing correlational analyses will be deposited to GitHub prior to publication, and the link will be provided at that time. Access to cell- and tissue-type heatmaps, as well as usage of cell- and tissue-type classification models, are available on reasonable request to academic investigators, without relevant conflicts of interest, for non-commercial use who agree not to distribute the data. Access requests can be made to: publications@pathai.com.
