# Supplementary material for: A feasibility study using quantitative and interpretable histological analyses of celiac disease for automated cell type and tissue area classification
Source: Sci Rep. 2024 Dec 2;14:29883. doi: 10.1038/s41598-024-79570-1 (PMC11612272; doi:10.1038/s41598-024-79570-1)
Supplement: Supplementary file 1 — Supplementary Information. [file 41598_2024_79570_MOESM1_ESM.docx]

Appendix. SUPPLEMENTAL MATERIAL

A feasibility study using quantitative and interpretable histological analyses of coeliac disease for automated cell type and tissue area classification

Michael Griffin^1^, Aaron M. Gruver^2^, Chintan Shah^1^, Qasim Wani^1^, Darren Fahy^1^, Archit Khosla^1^, Christian Kirkup^1^, Daniel Borders^1^, Jacqueline A. Brosnan-Cashman^1^, Angie D. Fulford^2^, Kelly M. Credille^2^, Christina Jayson^1^, Fedaa Najdawi^1,^*, Klaus Gottlieb^2,^*

* Fedaa Najdawi and Klaus Gottlieb contributed equally as co-senior authors.

AFFILIATIONS

^1^ PathAI, Boston, MA, USA

^2^ Eli Lilly and Company, Indianapolis, IN, USA

CORRESPONDING AUTHOR

Fedaa Najdawi

PathAI, Inc.

1325 Boylston Street, Suite 10000

Boston, MA 02215

USA

Tel: +1-617-500-8457, Email: [fedaa.najdawi@pathai.com](mailto:fedaa.najdawi@pathai.com)

LIST OF tables and FIGURES

**Supplemental Table 1** Correlations of model-derived HIFs with modified Marsh score.

**Supplemental Fig. 1** Workflow diagram of model development.

**Supplemental Fig. 2** (A) Data quality control and qualitative review of tissue and cell models to ensure exclusion of artifact regions (middle panel) and labelling of tissue regions (right panel). (B) Representative H&E-stained images and corresponding tissue overlays from normal duodenum and regions with moderate and severe villous blunting.

**Supplemental Fig. 3** Performance of cell predictions (frames) by the cell model.

Supplemental Table 1 Correlations of model-derived HIFs with modified Marsh score.

| **Feature** | **Spearman correlation coefficient** | ***P* value** |
| --- | --- | --- |
| Area proportion of villous epithelium over lamina propria in tissue | −0.836106 | <0.0001 |
| Area proportion of villous epithelium over mucosa in tissue | −0.788289 | <0.0001 |
| Area proportion of villous epithelium over all epithelium in tissue | −0.707178 | <0.0001 |
| Area proportion of villous epithelium over crypt epithelium in tissue | −0.707178 | <0.0001 |
| Area proportion of crypt epithelium over all epithelium in tissue | 0.707178 | <0.0001 |
| Area proportion of lamina propria over mucosa in tissue | 0.805047 | <0.0001 |
| Count proportion of intraepithelial lymphocytes over enterocytes in villous epithelium | 0.433375 | <0.0001 |
| Density of intraepithelial lymphocytes in villous epithelium | 0.527404 | <0.0001 |
| Density of plasma cells in mucosa | 0.587203 | <0.0001 |
| Count proportion of plasma cells over all cells in mucosa | 0.586244 | <0.0001 |
| Count proportion of eosinophils over all cells in mucosa | 0.594639 | <0.0001 |
| Density of eosinophils in mucosa | 0.612484 | <0.0001 |
| Count proportion of lymphocytes over plasma cells in mucosa | −0.639687 | <0.0001 |

HIF, human interpretable feature.

**
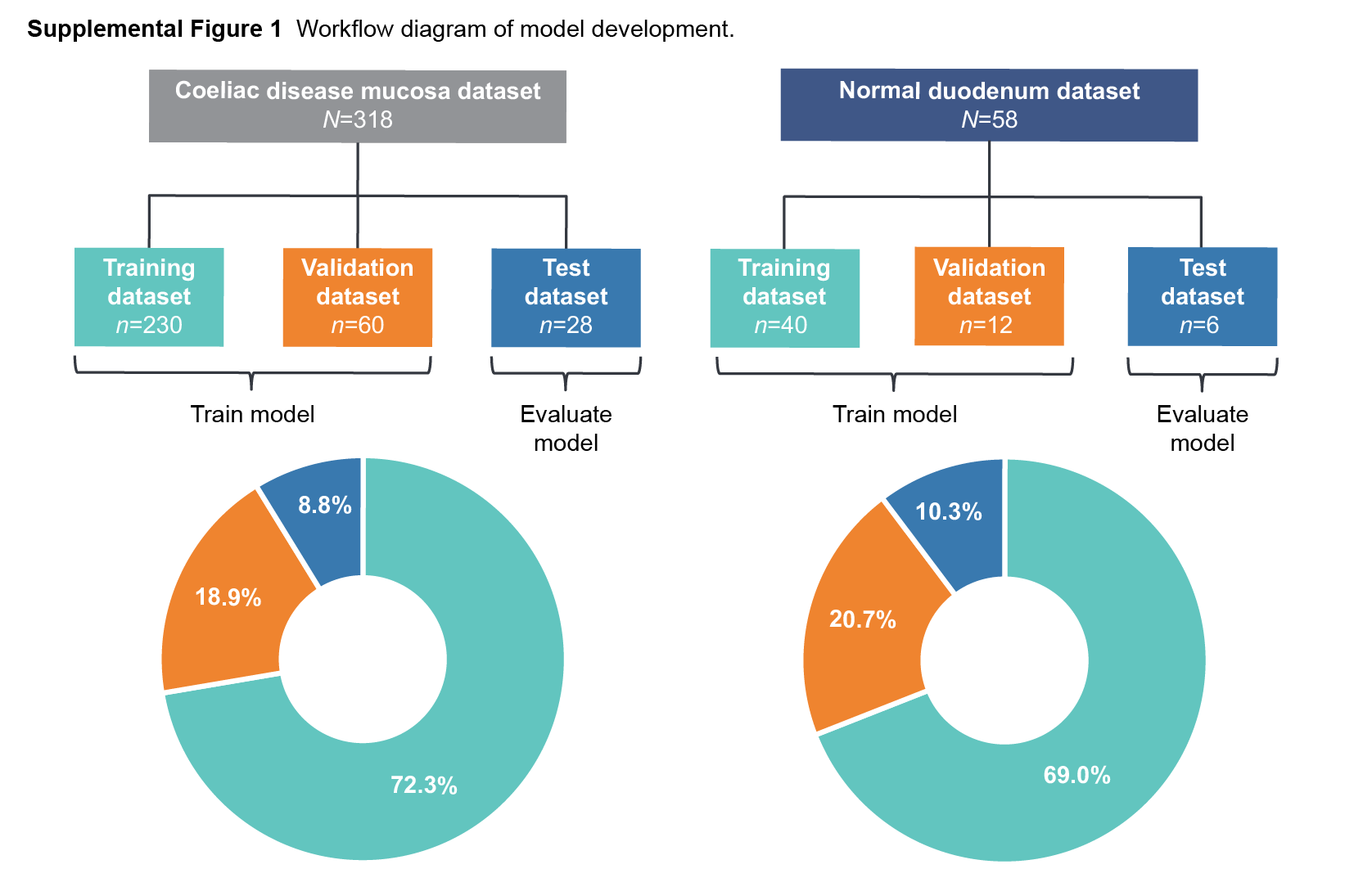
**

**Supplemental Fig. 1** Workflow diagram of model development.

**
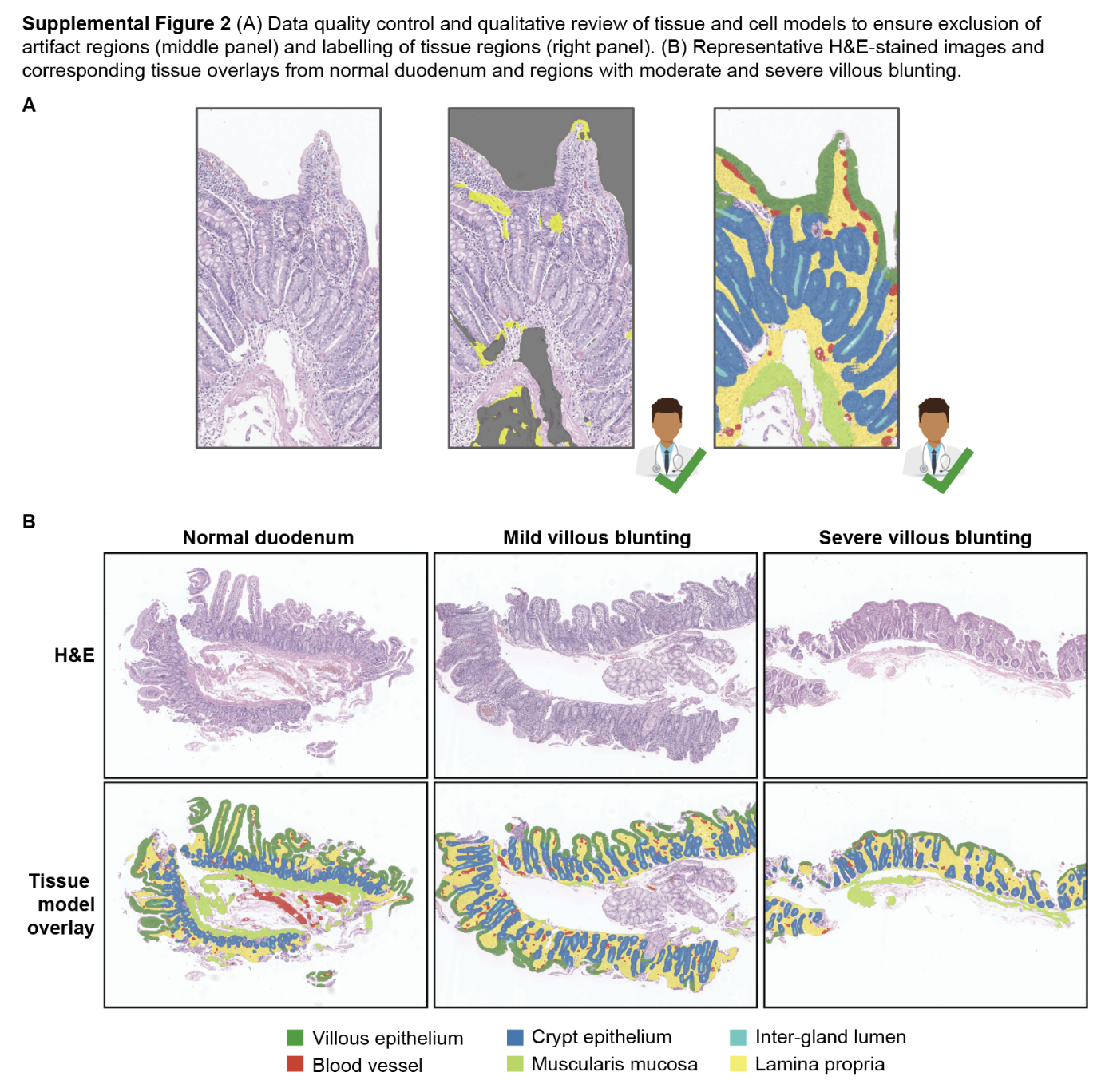
**

**Supplemental Fig. 2** (a) Data quality control and qualitative review of tissue and cell models to ensure exclusion of artifact regions (middle panel) and labelling of tissue regions (right panel). (b) Representative H&E-stained images and corresponding tissue overlays from normal duodenum and regions with moderate and severe villous blunting. H&E, haematoxylin and eosin.

**
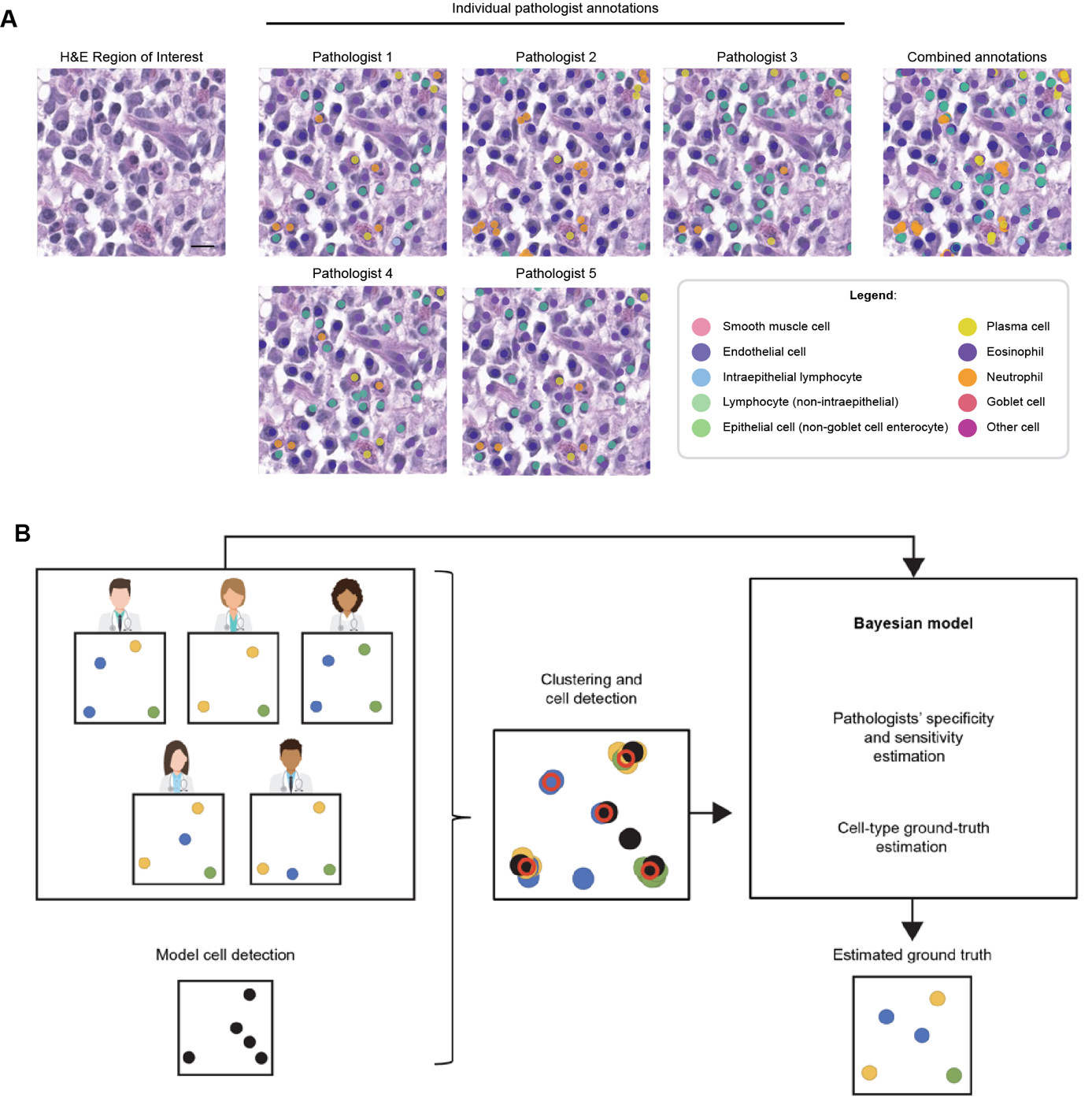
**

**Supplemental Fig. 3** A) Example annotations of individual cell types on a region (frame) of an H&E-stained WSI. Annotations from individual pathologists and all pathologists combined are shown. Scale bar indicates 10 μm. B) Performance of cell predictions (frames) by the cell model. Hierarchical clustering was performed on 1) annotations collected by pathologists (*n*=5) and 2) cell locations predicted by the cell model to locate true cells. A Bayesian model was then run using pathologist annotations as the input to estimate the ground truth cell type for each of the cell locations based on the estimated specificity and sensitivity of each annotator. Model predictions and pathologist predictions are then compared to that ground truth.
